# Method for correcting the temperature dependence of field-type glass electrode pH sensors

**DOI:** 10.1007/s44211-026-00948-4

**Published:** 2026-07-21

**Authors:** Kei Okamura, Takuroh Noguchi, Mayumi Hatta

**Affiliations:** 1 Faculty of Agriculture and Marine Science, Kochi University, B200, Monobe, Nankoku, Kochi 783-8502, Japan; 2 Usa Marine Biological Laboratory, Kochi University, 194, Inoshiri, Usa, Tosa, Kochi 781-1164, Japan

**Keywords:** Calibration for temperature, Glass electrode, Nernst response factor, pH sensor, Seawater

## Abstract

Seawater pH measurement is essential for monitoring ocean acidification and its biogeochemical impacts. Glass electrode pH sensors are widely used for in situ applications due to their ease of operation, low power consumption, and no reagent requirements. However, their accuracy can be affected by temperature-dependent effects, particularly those associated with the internal reference solution. Conventional calibration methods based on the Nernst response do not explicitly account for temperature-induced variations in the internal solution, which may lead to systematic bias under conditions with large temperature gradients. In this study, a correction method is proposed to address temperature-dependent errors by modeling the internal pH (pH_in_). The model incorporates two primary temperature-dependent contributions: the intrinsic temperature dependence of the phosphate buffer and potential shifts caused by activity changes in saturated KCl. These effects are combined through the electrode response factor (*f*_R_), defined as the ratio of the observed electrode slope to the theoretical Nernst slope, to estimate pH_in_ as a function of temperature and correct measured pH values to a reference temperature of 25 °C. Application of the method to vertical seawater profiles demonstrated a reduction in deviation from colorimetric reference measurements. The corrected results showed reduced temperature-dependent bias across depth. This approach provides a practical framework for improving the reliability of in situ glass electrode pH measurements under varying thermal conditions. The proposed method addresses equilibrium temperature-dependent effects of the internal solution but does not explicitly account for transient hysteresis, pressure effects, or long-term sensor drift.

**Graphical abstract:**

**Supplementary Information:**

The online version contains supplementary material available at https://doi.org/10.1007/s44211-026-00948-4.

**Figure:**
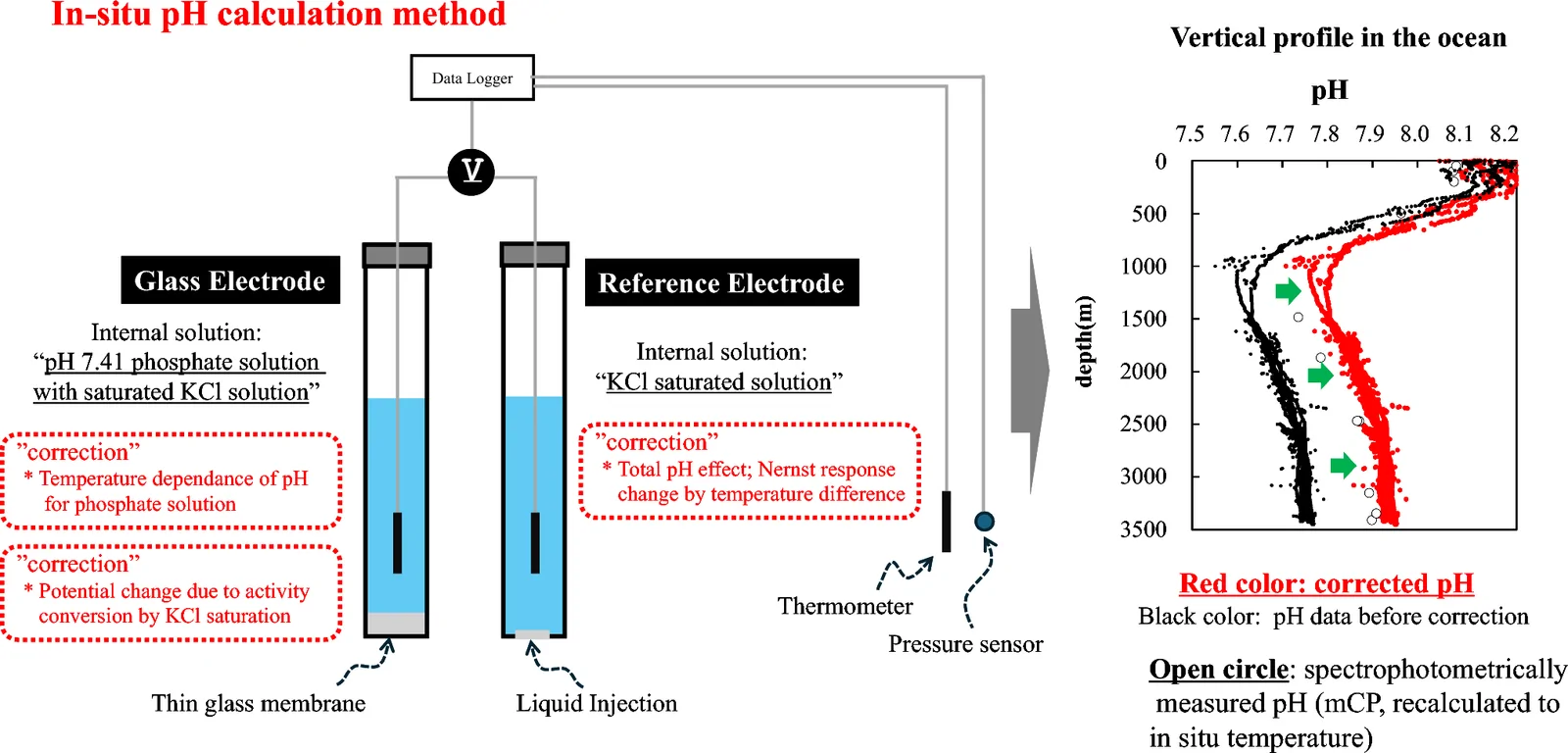

## Introduction

Seawater pH measurement has become an essential tool to directly monitor ocean acidification and evaluate its biogeochemical consequences. Glass electrode pH sensors are widely employed owing to their ease of operation and suitability for in situ applications. pH measurement using glass membrane electrodes consumes minimal power and requires no reagents, making it suitable for various underwater sensor applications. However, their accuracy is influenced by temperature-dependent effects, particularly those associated with the internal reference solution. Furthermore, the low stability and complex calibration of these electrodes pose challenges to pH measurement [1].

Although standard calibration procedures account for temperature through the Nernst response, the temperature-dependent variation in the internal solution’s composition has not been explicitly quantified or corrected. This omission can introduce systematic errors into pH measurements, especially under conditions of large temperature gradients encountered during vertical hydrocast observations.

We have estimated the pH change of the internal liquid of the electrode (assumed as a phosphate buffer) under saturated and unsaturated conditions of the coexisting KCl in the buffer for pH glass electrode calibration [2]. Building on these results, a correction method for temperature-dependent errors by explicitly modeling the variation in internal pH (pH_in_) has been proposed in this study. The approach is based on an electrochemical framework that incorporates buffer chemistry and electrochemical effects.

## Experimental section

### Reagents

Seawater-based pH buffers, comprising 2-amino-2-hydroxymethyl-1,3-propanediol (TRIS; catalog number 283-77321) and 2-aminopyride (AMP; catalog number 284-77331) dissolved in synthetic seawater (salinity: 35), were purchased from Wako. Pure-water-based buffers (pH = 6.86 and 7.41 at 25 °C) were purchased from Wako (Japan Calibration Service System; JCSS grade). The pH values of the buffers are presented in Supporting Information Table S1. Reagent-grade potassium chloride was also purchased from Wako. All solutions were prepared using Millipore Milli-Q Water.

### Theoretical aspects of pH measurement

pH measurement with a glass electrode is performed by recording the potential difference between the glass and reference electrodes. The measurement procedure is described in detail elsewhere [2]; only a brief summary is given here. The electrochemical voltage *E*_emf_ of the pH electrode [3] is expressed as.

1 $$ E_{{{\mathrm{emf}}}} = b - E_{{\mathrm{j}}} + 2.303\frac{RT}{F}\left( {{\mathrm{pH}}_{{{\mathrm{in}}}} - {\mathrm{pH}}_{{\mathrm{X}}} + a} \right), $$

2 $$ a = {\mathrm{log}}\frac{{a\left( {{\mathrm{Cl}}^{ - }_{{{\mathrm{ref}}}} } \right)}}{{a\left( {{\mathrm{Cl}}^{ - }_{{{\mathrm{in}}}} } \right)}}, $$

where *R* (= 8.3145 J mol^− 1^ K^− 1^) is the universal gas constant, *T* is the absolute temperature (K), *F* (= 96,485 C mol^− 1^) is Faraday’s constant, pH_in_ is the pH of the internal solution for the measuring glass electrode, pH_X_ is the pH of the sample solution, *E*_j_ is the liquid junction potential between the reference electrode and the sample solution, and *b* is a constant. *a*(Cl^−^_in_) and *a*(Cl^−^_ref_) are the chloride ion activities in the measuring and reference electrodes, respectively. The term 2.303(*RT*/*F*) in Eq. (1) is the Nernst factor *k* [4], which is proportional to the absolute temperature and is given by.

3 $$ k\left( {25^\circ {\mathrm{C}}} \right) = 2.303R \times \frac{273.15 + 25.00}{F} = 0.059160, $$

4 $$ k\left( t \right) = 0.059160 \times \left( {273.15 + t} \right)/\left( {273.15 + 25.00} \right), $$

where *k*(25 °C) and *k*(*t*) represent the Nernst responses of (V/pH) at 25 °C and *t*°C, respectively. Equation (1) shows that *E*_emf_ is proportional to the difference between pH_X_ and pH_in_. Because the measuring and reference electrodes are filled with concentrated KCl to maintain a fixed or saturated Cl^−^ concentration [5], *a* in Eq. (2) is effectively constant, and Eq. (1) simplifies to

5 $$ E_{{{\mathrm{emf}}}} = b - E_{{\mathrm{j}}} + k\left( t \right)\left\{ {{\mathrm{pH}}_{{{\mathrm{in}}}} - {\mathrm{pH}}_{{\mathrm{X}}} + a} \right\}. $$

In Eq. (5), *E*_j_ depends on electrode characteristics and may vary between measurements, whereas pH_in_ is determined by the composition of the internal solution and remains unchanged upon electrode replacement.

In practice, pH is determined by sequentially measuring *E*_emf_ at temperature *t* (°C) in two standard buffers of defined pH and in the sample solution. When all measurements are carried out at the same *t*, the operational pH of the sample is defined as:

6 $$ {\mathrm{pH}}_{{\mathrm{x}}} = {\mathrm{pH}}_{0} + E_{{{\mathrm{emf}},{\text{ x}}}} /s\left( t \right), $$

7 $$ s\left( t \right) = \left( {E_{{{\mathrm{emf}},{\mathrm{S}}2}} - E_{{{\mathrm{emf}},{\mathrm{S}}1}} } \right)/\left( {{\mathrm{pH}}_{{{\mathrm{S}}2}} - {\mathrm{pH}}_{{{\mathrm{S}}1}} } \right), $$

8 $$ {\mathrm{pH}}_{0} = {\mathrm{pH}}_{{{\mathrm{S}}1}} - E_{{{\mathrm{emf}},{\text{ s}}1}} /s\left( t \right), $$

where subscripts X, S1, and S2 represent the sample and standard buffers, respectively; pH_0_ is the pH when *E*_emf_ =  0 mV; and *s*(*t*) is the slope of the calibration line at temperature *t* [4]. Notably, this calculation requires neither *b*, *E*_j_, *a*, nor pH_in_.

The response factor *f*_R_ at temperature *t*°C is obtained from *s*(*t*) in Eq. (7) and *k*(*t*) in Eq. (4) as:

9 $$ f_{{\mathrm{R}}} = - s\left( t \right)/k\left( t \right). $$

when the electrode exhibits an ideal Nernst response, *f*_R_ equals 1; as the response deteriorates, the value of *f*_R_ decreases.

### *In situ* glass electrode pH sensor (PH-09)

An in situ glass electrode pH sensor (PH-09) was employed to measure the pH of seawater samples. A detailed description of PH-09 is provided in a previous study [6]; its configuration can be summarized as follows. PH-09 comprises two electrodes: (i) a glass electrode with a pH-sensitive glass membrane and (ii) a reference electrode maintained under saturated AgCl. The glass electrode contains a sealed pH 7.41 phosphate standard solution serving as the internal pH reference. KCl is dissolved in this phosphate solution to saturation, and KCl powder is sealed within the electrode to ensure that the solution remains saturated regardless of temperature changes. The reference electrode is similarly sealed with KCl powder, rendering it a KCl-saturated solution that is equivalent to the internal solution of the measuring glass electrode.

In situ measurements using PH-09 were conducted in the northwest Pacific (27.9 °N, 140.7 °E) during the R/V Hakuho-Maru (JAMSTEC) KH-11-5 cruise. Depth and temperature were measured using a conductivity, temperature and depth (CTD) carousel-mounting system with water samplers (SBE 911 Plus, Seabird). PH-09 was calibrated onboard using the TRIS and AMP buffers, both adjusted to 25 °C. The *E*_emf_ of the pH cell and liquid temperature of the two buffers were measured. The pH values of the two buffers at the measured temperatures were calculated based on a study conducted by Dickson et al. [4].

## Results and discussion

### pH temperature-dependent correction method

Although Eq. (6) shows that pH_in_ does not need to be considered, at the calibration temperature, Eq. (1) indicates that the electrode potential is a function of pH_in_. The following analysis divides this effect into two contributions: (i) the intrinsic temperature dependence of the pH 7.41 phosphate buffer, (ii) the potential shift arising from activity conversion in the saturated KCl solution. These two effects are combined through the electrode response factor (*f*_R_) to estimate pH_in_ as a function of temperature and correct measured pH values to a reference temperature of 25 °C.

### Temperature dependence of the phosphate buffer pH

**Fig. 1:**
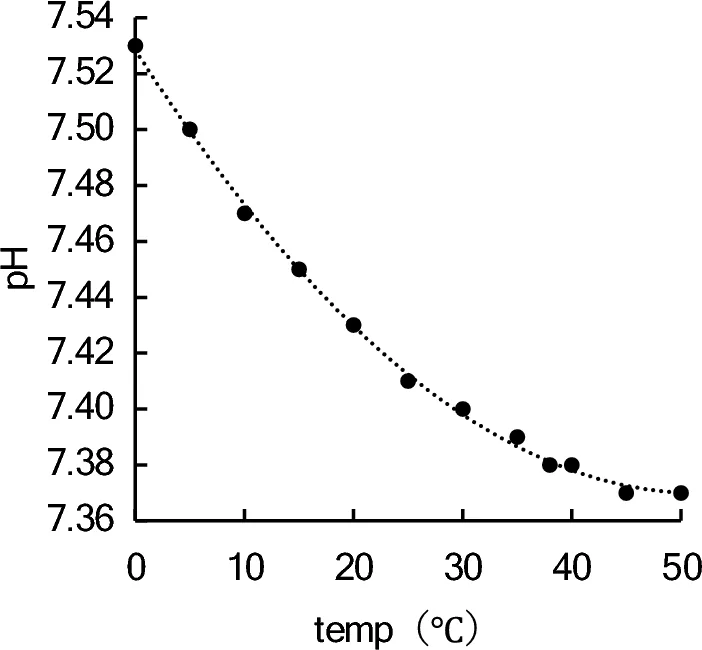
Temperature dependence of the pH of the phosphate buffer solution. Dotted line: approximation curve from Eq. (10)

The internal solution of PH-09 consists of a pH 7.41 phosphate buffer. The pH values of this solution as a function of temperature are listed in Supporting Information Table S1. The temperature dependence of the phosphate-buffer component can be described by the following quadratic regression:

10 $$ {\mathrm{pH}}_{{{\mathrm{in}}\_{\mathrm{soln}}}} \left( t \right) = 0.00005892 \times t^{2} - 0.006115 \times t + 7.528, $$

where *t* is the water temperature (°C.) Fig. 1 shows an approximation curve reproduced from the tabulated values in Table S1. Equation (10) represents the temperature dependence of the phosphate-buffer component only. The effect of coexisting saturated KCl is treated separately in the following section and incorporated into the final correction model.

### Potential change due to activity conversion by saturated KCl

The *E*_emf_ of the system is influenced by the KCl concentration coexisting with the phosphate buffer, and this influence varies with temperature. The *E*_emf_ shift at 25 °C and *t*°C, denoted as *E*_chg_(25 °C) and *E*_chg_ (*t*), respectively, can be estimated from the KCl molality *m*_KCl_ using the following relations [2]:

11 $$ E_{chg} \left( {25^\circ {\mathrm{C}}} \right) = - 0.6248 \times m_{{{\mathrm{KCl}}}}^{2} + 6.4917 \times m_{{{\mathrm{KCl}}}} + {14}.{451}, $$

12 $$ E_{chg} \left( t \right) = E_{chg} \left( {25^\circ {\mathrm{C}}} \right) \times k\left( t \right)/k\left( {25^\circ {\mathrm{C}}} \right). $$

The molality of saturated KCl, *m*_KCl,sat_ (mol/kg-H_2_O), at temperature *t* (°C) is approximated by the following equation [2]:

13 $$ m_{{{\mathrm{KCl}},{\mathrm{sat}}}} \left( t \right) = 0.04116t + 3.7334. $$

### Total pH effect

Combining the two contributions above, the total internal pH pH_in_(*t*) of the internal solution at *t* (°C) is expressed as:

14 $$ {\mathrm{pH}}_{{{\mathrm{in}}}} \left( t \right) = {\mathrm{pH}}_{{{\mathrm{in}}_{{{\mathrm{soln}}}} }} \left( t \right) - E_{{{\mathrm{chg}}}} \left( t \right)/\left\{ {f_{{\mathrm{R}}} \times k\left( t \right)} \right\}. $$

**Fig. 2:**
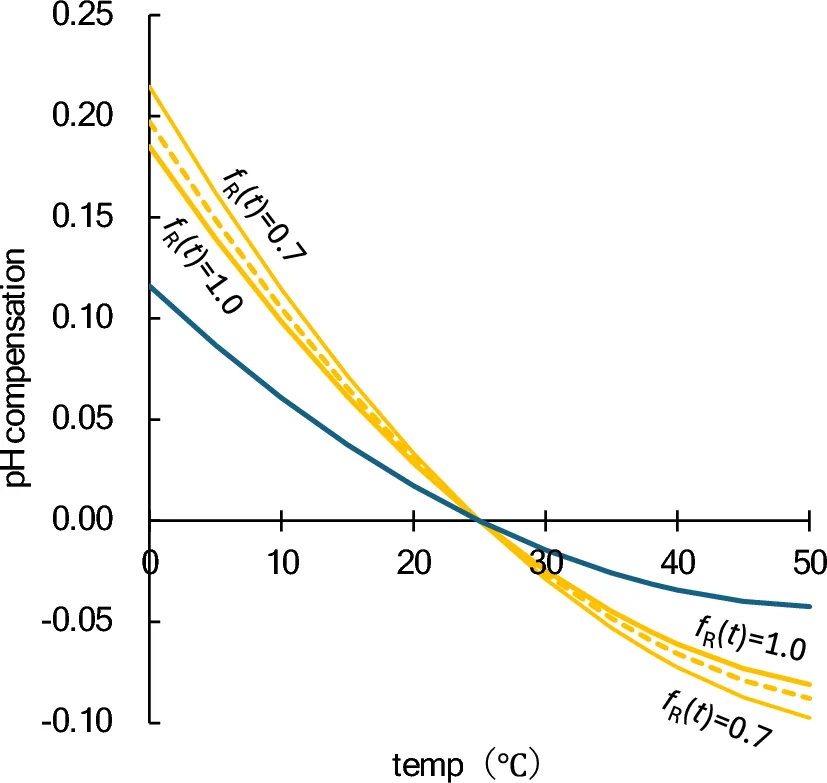
Departure of pH_in_(*t*) from the reference value at 25 °C as a function of temperature. Blue line: contribution from pH_in_soln_(*t*) alone; orange lines: total pH_in_(*t*) departure; solid lines: *f*_R_ = 1.0 (100% Nernst response) and 0.7 (70%); dotted line: *f*_R_ = 0.85 (85%)

Figure 2 illustrates the departure of pH_in_(*t*) from its value at 25 °C as a function of temperature. The results show that pH_in_(*t*) increases as temperature decreases and vice versa. The magnitude of this effect grows as *f*_R_ departs from unity, i.e., as electrode performance degrades. As *f*_R_ decreases, i.e., as the response of the electrodes deteriorates, the temperature effect intensifies.

### Compensation of PH-09 data to water temperature

#### Calculation method

Since pH_in_ is temperature-dependent as expressed by Eq. (14), the electrode potential recorded during sample measurement is correspondingly affected, as indicated by Eq. (5). The resulting pH error can be removed by referencing pH_in_(*t*) to its value at 25 °C using the following equation:

15 $$ {\mathrm{pH}}_{{{\mathrm{comp}}}} \left( t \right) = {\mathrm{pH}}_{{{\mathrm{dev}}}} \left( t \right) + {\mathrm{pH}}_{{\mathrm{X}}} \left( t \right), $$

16 $$ {\mathrm{pH}}_{{{\mathrm{dev}}}} \left( t \right) = {\mathrm{pH}}_{{{\mathrm{in}}}} \left( t \right) - {\mathrm{pH}}_{{{\mathrm{in}}}} \left( {25^\circ {\mathrm{C}}} \right), $$

where pH_comp_(*t*) is the temperature-corrected pH and pH_dev_ (*t*) is the pH deviation at *t*°C relative to 25 °C.

### Sample data calculation

**Fig. 3:**
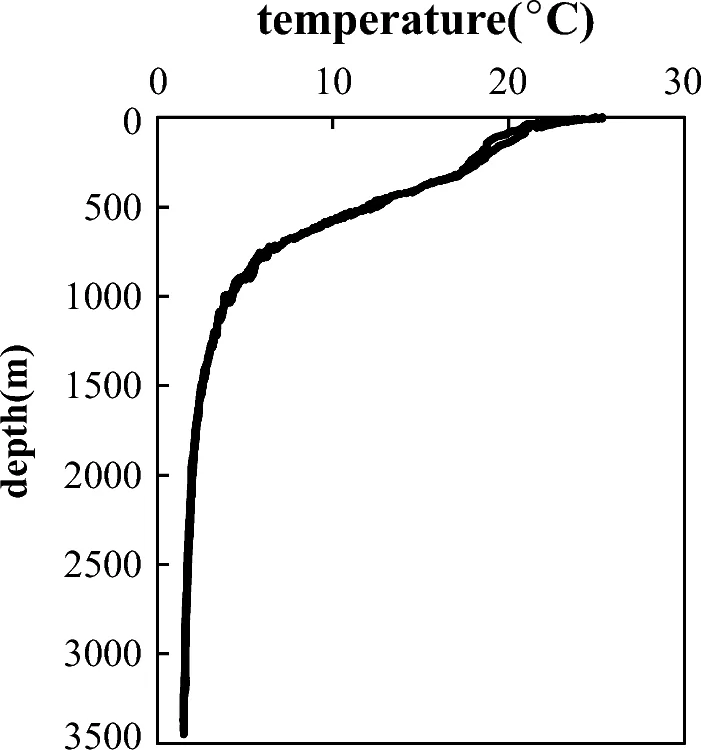
Vertical profile of water temperature using data reported by Okamura et al. [2]

**Fig. 4:**
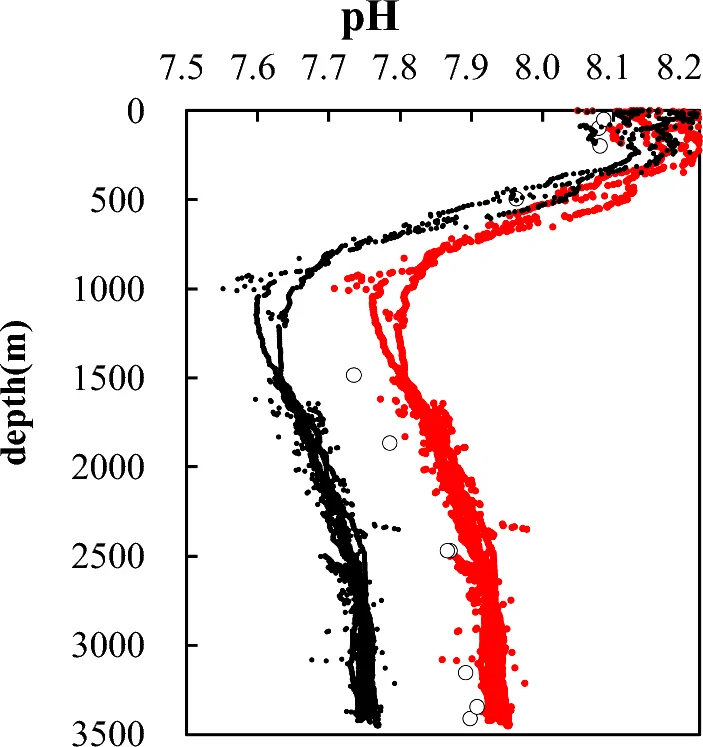
Vertical profiles of pH. Open circles: pH measured by shipboard colorimetric analysis of water samples and recalculated to in situ water temperature reported by Okamura et al. [7]. Black dots: uncorrected PH-09 values reported by Okamura et al. [2]. Red dots: corrected values from Eq. (15)

Equation (15) was applied to recalculate the vertical pH profile reported by Okamura et al. [6]. The calibrated values of *f*_R_ and pH_0_ were 0.850 and 6.870, respectively [6]. The corresponding vertical temperature profile is shown in Fig. 3, and a comparison of the corrected and uncorrected pH profiles is displayed in Fig. 4. Relative to the open-circle reference values obtained by colorimetric analysis of the collected water samples [7], the uncorrected profile (black dots) exhibits a large negative bias that increases with depth.

Table 1 presents the results of the numerical verification. Deviations from colorimetric pH are listed for depths ranging from 50 to 3410 m. At shallow depths (50–500 m), both uncorrected and corrected pH values show positive deviations from the colorimetric pH, and in some cases the deviation becomes larger after correction. In contrast, at depths greater than approximately 1500 m, the corrected values are substantially closer to the colorimetric pH than the uncorrected values. In the uncorrected results, the deviation changes systematically from positive values at shallow depths (high temperatures) to negative values at deep depths (low temperatures). After applying the correction, this temperature-dependent trend is greatly reduced, and the residual deviations remain consistently positive with little dependence on depth. The present correction addresses the equilibrium temperature dependence of the internal solution and does not explicitly compensate for thermal hysteresis under rapidly changing temperature conditions. These results indicate that the proposed method effectively compensates for the systematic bias associated with the temperature dependence of the internal solution.

**Table 1:** Comparison between colorimetric pH and PH-09 pH values before and after temperature correction

Depth (m)	Colorimetric In situ pH^a^	Uncorrected In situ pH^b^	Deviation^c^	Corrected pH In situ pH^d^	Deviation^c^
50	8.086	8.201	+ 0.115	8.227	+ 0.141
100	8.079	8.148	+ 0.069	8.182	+ 0.103
200	8.081	8.154	+ 0.073	8.196	+ 0.115
500	7.963	8.026	+ 0.063	8.113	+ 0.150
1485	7.735	7.630	− 0.105	7.804	+ 0.069
1865	7.885	7.682	− 0.103	7.860	+ 0.075
2470	7.870	7.720	− 0.150	7.901	+ 0.031
3155	7.892	7.732	− 0.160	7.916	+ 0.024
3345	7.908	7.746	− 0.162	7.930	+ 0.022
3410	7.898	7.742	− 0.156	7.925	+ 0.027

^a^Data reported by Okamura et al. [7]; ^b^Black dot data shown in Fig. 4 for upcast; ^c^Deviation from colorimetric pH; ^d^Red dot data shown in Fig. 4 for upcast. Sample collection was performed at the time the sensor was brought up onto the ship; therefore, the extracted sensor data correspond to the same time as that of sample collection

Although the composition of the internal solution may differ among manufacturers, the proposed framework can be applied when the temperature dependence of the internal solution is known or can be reasonably estimated and *f*_R_ can be determined or assumed. For example, if the internal solution is based on a phosphate buffer, a buffer solution with pH 6.86 exhibits the same temperature-dependent trend as a buffer solution with pH 7.41 between 0 °C and 35 °C (Table S1), and therefore the same correction framework can be applied.

Values corrected using this method generally show small positive deviations. This is presumed to be due to factors unrelated to the temperature dependence of the internal solution, such as changes in the asymmetric potential caused by pre-calibration conditions. Therefore, maintaining the sensor in a hydrated state, avoiding direct sunlight exposure, and minimizing the time interval between calibration and deployment may improve measurement accuracy. The present model considers temperature-dependent effects of the internal solution only. Possible influences of hydrostatic pressure on the internal solution and electrode response, as well as variations in seawater composition, particularly salinity, that may affect the liquid junction potential (*E*_j_), were not evaluated in this study and are therefore beyond the scope of the present correction framework.

## Conclusion

Temperature-dependent changes in the internal solution represent a major source of error in field-type glass electrode pH sensors. A correction method was developed by considering two primary temperature-dependent contributions: the intrinsic temperature dependence of the phosphate buffer pH and the potential shift due to activity conversion in saturated KCl. These effects are combined through the electrode response factor (*f*_R_) to estimate pH_in_ as a function of temperature and correct measured pH values to a reference temperature of 25 °C. Application of this method to vertical seawater profiles, where temperature varies considerably with depth, demonstrated a reduction in temperature-dependent bias compared to colorimetric references.

These results indicate that the proposed correction method reduces systematic errors associated with the temperature dependence of the internal solution during in situ glass electrode pH measurements under variable temperature conditions. Although the composition of the internal solution may differ among manufacturers, the proposed framework can be applied when the temperature dependence of the internal liquid is known or can be reasonably estimated and *f*_R_ can be determined or assumed. The method may be applicable to vertical ocean observations from shallow to deep waters, such as those obtained from Argo floats [8], provided that sensor calibration history and operational conditions are appropriately considered.

## Supplementary Information

Below is the link to the electronic supplementary material.

Supplementary file1 (DOCX 25 kb)

## Data Availability

The datasets generated and analyzed during the current study are available from the corresponding author upon reasonable request.

## References

[1] V.M. Rérolle, C.F.A. Floquet, M.C. Mowlem, R.R.G.J. Bellerby, D.P. Connelly, TrAC Trends. Anal. Chem. (2012). 10.1016/j.trac.2012.07.016

[2] K. Okamura, T. Noguchi, M. Hatta, Galaxea J Coral Reef Stud. (2025). 10.3755/galaxea.G27O-4

[3] S. Ben-Yaakov, E. Ruth, Limnol. Oceanogr. (1974). 10.4319/lo.1974.19.1.0144

[4] A.G. Dickson, C.L. Sabine, J.R. Christian. (PICES Special Publication 3, USA, 2007)

[5] K. Schwabe, H.D. Suschke, Angew Chem Int Ed English. (1964). 10.1002/anie.196400361

[6] K. Okamura, T. Noguchi, M. Hatta, J. Ishibashi, J, In 2016 Techno-Ocean (Techno-Ocean), Kobe, Japan. IEEE. (2016). 10.1109/Techno-Ocean.2016.7890688

[7] K. Okamura, H. Kimoto, T. Noguchi, M. Hatta, H. Kawakami, T. Suzue, Anal. Sci. (2014). 10.2116/analsci.30.1135 25492462

[8] W.B. Owens, N. Zilbterman, K.S. Johnson, H. Claustre, M. Scanderbeg, M.S. Wijffels, T. Suga, Mar. Technol. Soc. J. (2022). 10.4031/MTSJ.56.3.8

