# Supplementary material for: Method for correcting the temperature dependence of field-type glass electrode pH sensors
Source: Anal Sci. 2026 Jul 21;42(10):1027–32. doi: 10.1007/s44211-026-00948-4 (PMC13588918; doi:10.1007/s44211-026-00948-4)
Supplement: Supplementary file 1 [file 44211_2026_948_MOESM1_ESM.docx]

**Method for correcting the temperature dependence of field-type glass electrode pH sensors**

Analytical Sciences

Kei OKAMURA*^1^, Takuroh NOGUCHI^1^, and Mayumi HATTA^1^

Address: ^1^Interdisciplinary Science Unit, Multidisciplinary Science Cluster, Research and Education Faculty, Kochi University

Usa Marine Biological Laboratory, 194, Inoshiri, Usa, Tosa, Kochi 781-1164, Japan

* Corresponding author

Email: Kei OKAMURA – okamurak@kochi-u.ac.jp

**Table S1:** pH values of the standard pH solutions provided by Wako

| temp(°C) | pH 7.41 buffer solution | | pH 6.86 buffer solution | |
| --- | --- | --- | --- | --- |
|  | pH value | difference from 25 (°C) | pH value | difference from 25 (°C) |
| 0 | 7.53 | +0.12 | 6.98 | +0.12 |
| 5 | 7.50 | +0.09 | 6.95 | +0.09 |
| 10 | 7.47 | +0.06 | 6.92 | +0.06 |
| 15 | 7.45 | +0.04 | 6.90 | +0.04 |
| 20 | 7.43 | +0.02 | 6.88 | +0.02 |
| 25 | 7.41 | --- | 6.86 | --- |
| 30 | 7.40 | −0.01 | 6.85 | −0.01 |
| 35 | 7.39 | −0.02 | 6.84 | −0.02 |
| 38 | 7.38 | −0.03 | 6.84 | −0.02 |
| 40 | 7.38 | −0.03 | 6.84 | −0.02 |
| 45 | 7.37 | −0.04 | 6.83 | −0.03 |
| 50 | 7.37 | −0.04 | 6.83 | −0.03 |
